# Critical controllability analysis of directed biological networks using efficient graph reduction

**DOI:** 10.1038/s41598-017-14334-8

**Published:** 2017-10-30

**Authors:** Masayuki Ishitsuka, Tatsuya Akutsu, Jose C. Nacher

**Affiliations:** 1 Department of Information Science, Faculty of Science, Toho University, Funabashi, 274-8510 Japan; 2 Bioinformatics Center, Institute for Chemical Research, Kyoto University, Uji, 611-0011 Japan

## Abstract

Network science has recently integrated key concepts from control theory and has applied them to the analysis of the controllability of complex networks. One of the proposed frameworks uses the Minimum Dominating Set (MDS) approach, which has been successfully applied to the identification of cancer-related proteins and in analyses of large-scale undirected networks, such as proteome-wide protein interaction networks. However, many real systems are better represented by directed networks. Therefore, fast algorithms are required for the application of MDS to directed networks. Here, we propose an algorithm that utilises efficient graph reduction to identify critical control nodes in large-scale directed complex networks. The algorithm is 176-fold faster than existing methods and increases the computable network size to 65,000 nodes. We then applied the developed algorithm to metabolic pathways consisting of 70 plant species encompassing major plant lineages ranging from algae to angiosperms and to signalling pathways from *C. elegans, D. melanogaster* and *H. sapiens*. The analysis not only identified functional pathways enriched with critical control molecules but also showed that most control categories are largely conserved across evolutionary time, from green algae and early basal plants to modern angiosperm plant lineages.

## Introduction

An understanding of the functional processes and mechanisms of living cells requires combined knowledge of multiple life molecules. However, these biological functions are not the result of single molecules but rather tend to emerge from the interactions among molecules. Complex diseases are not an exception; in fact, these diseases are often the result of failures in interactions and molecules that can exert effects on distant pathways^1,2^, and this view naturally leads to the concept of biological networks. To restore functional features or reverse a disease state, one prospective way is to apply intervention or control to the disrupted pathways and molecular processes. Since it is impossible to directly alter the states of all genes or molecules, it is needed to find a small number of nodes in a given network control of which drives the whole network to a desired state, where these kinds of nodes are called driver nodes.

Indeed, recent research has led to the development of several frameworks for this controllability problem in complex networks. Liu *et al*. employed the structural controllability framework for linear systems and showed that the minimum set of driver nodes can be found by computing the Maximum Matching (MM) in graph theory^3^. However, they also showed that this approach requires a large number of driver nodes in scale-free networks^3^. As an alternative approach, Nacher and Akutsu proposed the Minimum Dominating Set (MDS) approach^4^, where MDS is also a well-known concept in graph theory. They showed that the MDS approach needs a smaller fraction of nodes in scale-free networks. Since most of the observed complex networks in nature are scale-free networks and the MDS approach can cope with non-linear systems^4,5^, analysing the controllability of real networks using the MDS approach appears reasonable. Actually, the MDS approach has been applied to analysis of various biological networks and reported to be useful for finding important genes and molecules not only by our group but also by other groups^6–19^.

Although finding the MDS in a given network is known as a hard problem (NP-hard problem), the MDS can be found in reasonable CPU time in many practical cases by using Integer Linear Programming (ILP) if the network has a scale-free property. However, the MDS is not uniquely determined, that is, there might exist multiple minimum dominating sets. In order to cope with this non-uniqueness issue, the concepts of critical, intermittent and redundant nodes were applied to the MDS framework^16^, where these concepts were originally proposed for the MM framework^20^. Critical nodes are nodes included in all MDSs, whereas nodes that belong to some but not all MDSs are called intermittent, and those nodes that do not participate in any MDS are called redundant^16^. The computation of these control categories is very intense and requires solving the MDS problem *n* times, where *n* is the number of nodes. For undirected networks, in order to address this computational difficulty, a novel algorithm that benefits from heuristics pre-processing procedures has been proposed^10^. This algorithm was successfully used to investigate controllability properties by integrating a proteome-wide protein interaction network with transcriptome information.

However, many real-world networks, including metabolic pathways and signal pathways, are directed. To efficiently examine directed controllability features in these systems, we propose a new algorithm that includes heuristics to speed up the computation time and increase the computable network size. The performance of the algorithm was examined using artificial complex networks. The results showed that the algorithm expanded the computable size to 65,000 nodes and increased the computational speed 176-fold.

The significant improvement in performance obtained with the proposed algorithm allows the first directed MDS controllability analysis of large-scale directed biological networks, such as metabolic networks and signal pathways. The results provide novel insights and findings on specific functional pathways that are significantly enriched in critical control molecules and other control categories and show that most control categories are largely conserved across evolutionary time, from green algae and early basal plants to modern angiosperm plant lineages.

## Results

### Theoretical and computational analysis

#### Theoretical analyses of control categories led to an efficient MDS algorithm

The formal definition of an MDS in a directed network is as follow: Let *G(V*, *E)* be a directed graph, where *V* and *E* are sets of nodes and directed edges, respectively. Then, each node pair *(u, v)* ∈ *E* denotes a directed edge from *u* to *v*. In a directed network, *S (S* ⊆ *V)* is a Dominating Set (DS) if each node in *V* is an element of *S* or has directed edge(s) from one or more elements of *S* to the node. If the size of *S* is smallest among all existing DSs, it is called an MDS. The computation of an MDS is an NP-hard problem^21,22^. Therefore, the development of an algorithm that can solve the problem in polynomial time is not plausible. However, we formalised the MDS problem using Integer Linear Programming (ILP), in which ${x}_{v}=1$ and ${x}_{v}=0$ indicate that node *ν* is in an MDS and not in an MDS, respectively:

Minimise

$$\sum _{v\in V}{x}_{v}$$

subject to

1 $$\begin{array}{c}{x}_{v}+\sum _{(u,v)\in V}{x}_{u}\ge 1\,\forall v\in V,\\ {x}_{v}\in \{0,1\}\,\,\forall v\in V.\end{array}$$

Here, by solving the ILP (Eq. 1), an MDS is given by the set $\{v|{x}_{v}=1\}$.

It should be noted that an MDS satisfies the structural controllability condition, as demonstrated in Theorem 1 in our previous work^4^. However, this computational approach has some problems. First, the solution for a given MDS problem is not unique, which means that multiple node configurations might have the same minimum node size but contain different nodes. This problem is particularly serious when analysing real data. Previous studies defined control categories (critical, intermittent and redundant) to address this issue in both MM and MDS approaches^16,20^. A node is critical if it belongs to all MDS solutions. A node is considered intermittent if it belongs to at least one but not all MDS solutions, and a node is redundant if it does not belong to any MDS configuration. To identify these control categories, particularly the critical set of nodes, algorithms for undirected and directed networks have been proposed^16^. These algorithms successfully identify the control categories in complex networks. However, the network size that can be handled by these algorithms is limited to a few thousand nodes at most, and the computational time is not fast because the MDS problem needs to be solved *n* times, where *n* is the number of nodes. Therefore, pre-processing steps inspired by theoretical advances are necessary for the efficient computation of large networks consisting of tens of thousands of nodes.

Here, we present a new algorithm that computes controllability categories in directed networks using the MDS approach combined with efficient graph reduction techniques. The main novelty of the proposed method is the incorporation of heuristics in a pre-processing step to significantly improve the computation efficiency in terms of both computable size and computational speed. The details of the algorithm are shown in the Methods section. This manuscript also describes theoretical results obtained using the graph reduction process.

First, we invoke two important propositions for controllability in directed networks^16^:

Proposition 2.3 If *ν* is a node with indegree 0, *ν* is a critical node.

Proposition 2.4 If *ν* is a node with at least two directed edges to nodes with outdegree 0 and indegree 1, *ν* is a critical node.

These two propositions are related to the identification of critical nodes. However, a large fraction of redundant nodes could also be predetermined based on the following new proposition:

Proposition 1: If *ν* is a node with outdegree 0 and has an incoming link from a critical node, then *ν* is a redundant node.

**Figure 1:**
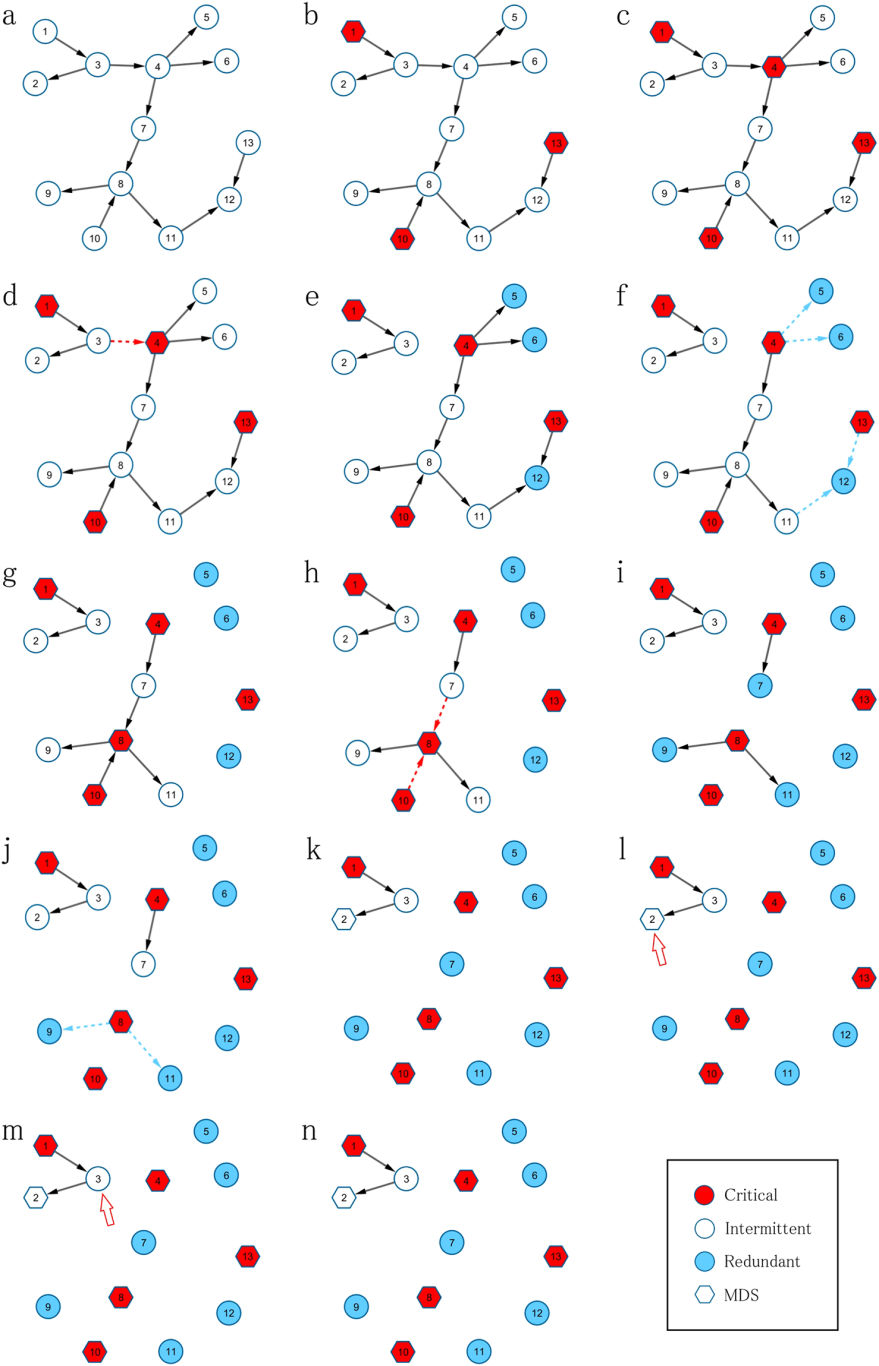
Illustration of the steps in the fast MDS algorithm. This graphical illustration follows the algorithm description shown in the Methods section. (**a**) Consider a directed graph of 13 nodes. (**b**) The application of proposition 2.3 to each node identifies a preliminary set of critical nodes. (**c**) Proposition 2.4 is then applied to each remaining unclassified node. (**d**) The incoming links to the newly identified critical node are deleted. (**e**) The application of proposition 1 to unclassified nodes leads to the identification of redundant nodes. (**f**) The incoming links to the newly identified redundant nodes are deleted. (**g–k**) Because links were deleted in previous steps, propositions 2.4 and 1 are re-applied to delete additional links. (**l**) ILP is used to find an MDS in the reduced graph, and the critical algorithmic procedure is applied to each node that belongs to the MDS and that has not already been identified as critical to determine its critical role. (**m**) The redundant algorithmic procedure is applied to each node that does not belong to the MDS to determine its redundant role. Both remaining nodes (2 and 3) are classified as intermittent.

**Figure 2:**
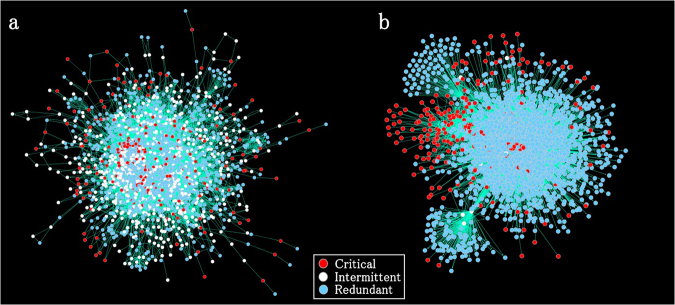
Network visualisation showing the classification of the nodes in a real plant metabolic network of *A. thaliana* (**a**) and in a signal network of *H. sapiens* (**b**) into control categories.

The combination of these three propositions led to the design of an efficient algorithm for the computation of control categories in large directed complex networks (see the Methods section). The operations performed by the algorithm are illustrated in Fig. 1, and an example of the classification of nodes in real metabolic and signalling pathway networks into control categories is shown in Fig. 2.

### Computational experiments using artificial networks

**Figure 3:**
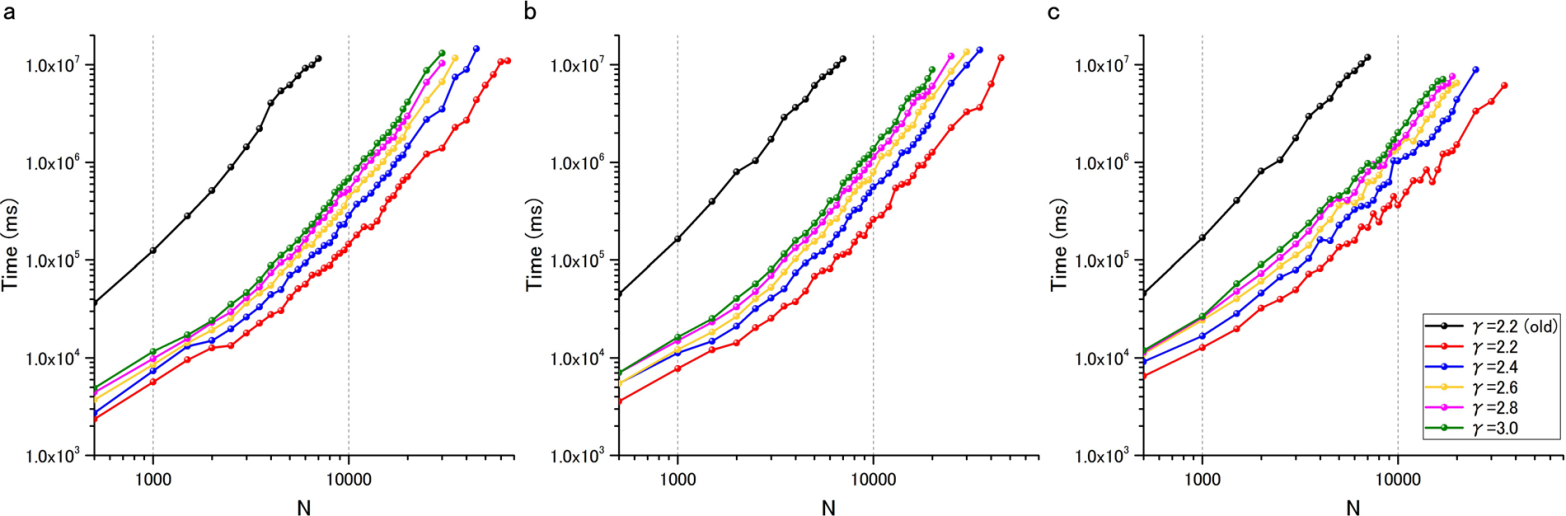
Results from the application of the MDS algorithm to artificially generated complex networks. The figure shows the computational time as a function of the network size *N* for scale-free networks with the following average degrees on log-log scale: (**a**) <k> = 2, (**b**) <k> = 2.5 and (**c**) <k> = 3.0. The results for networks with different degree exponents from *γ* = 2.2 to *γ* = 3.0 are shown in each figure. The colour legend for these values is shown in Fig. 3(c). Note that *γ* refers to ${\gamma }_{in}={\gamma }_{out}$ because only directed scale-free networks were considered.

To assess the performance of the algorithm, we constructed scale-free networks of different sizes, ranging from 500 to 65,000 nodes, with a degree distribution that follows a power law ${k}^{-\gamma }$. We also investigated the performance of the proposed algorithm when varying two topological parameters, such as the degree exponent *γ* and the average degree <*k*>. First, for each parameter pair, ten undirected scale-free networks were constructed using the Havel–Hakimi algorithm with random (Monte-Carlo) edge swaps (HMC model)^23^. The direction for each undirected link was then randomly selected, leading to the generation of directed networks with the same incoming and outgoing degree exponents ${\gamma }_{in}={\gamma }_{out}$. This procedure was repeated ten times, yielding a total of 100 networks for each data point. Figure 3(a–c) shows the average results obtained with the algorithm. The specific degree exponents examined were ${\gamma }_{in}={\gamma }_{out}$ = 2.2, 2.4. 2.6, 2.8, and 3.0, and the average degree values were <*k*> = 2, 2.5 and 3. Compared with our previous algorithm (black line), the proposed method (red line) increased the computation speed 176-fold, as determined using the computational time for N = 4,500 nodes with <*k*> = 2 as a reference. For a network size of N = 7,000, the computation speed was 156-fold faster than that of existing algorithms. Moreover, the computable network size was expanded to 65,000 nodes for some topologies. In general, we observed that increasing the average degree < *k* > from 2 to 3 increased the computational time (Fig. 3(a–c)) because the density of the edges increased, complicating the solution of the MDS. In contrast, scale-free networks with lower degree exponents were solved faster, suggesting the existence of high-degree nodes.

Although our new method significantly expanded the size of networks for which the critical, intermittent, and redundant nodes can be obtained, the MDS problem is NP-hard and thus exponential time is still needed in the worst case. Indeed, the log-log plots (see Fig. 3) suggest this tendency because if the computation time were polynomial, the curves would be almost straight. Therefore, it is reasonable that there exists some limit on the size of available networks. However, if much faster ILP solvers are developed in the future, larger networks might be handled. Since ILP solvers have been continuously improved, we can expect such improvements. Furthermore, when ${\gamma }_{in}={\gamma }_{out}$ = 2.2 is small, our method could compute the critical, intermittent, and redundant nodes for large networks with 65,000 nodes.

### Analysis of directed biological networks

In this study, we applied the proposed efficient MDS algorithm to directed biological networks such as metabolic and signalling pathways. For metabolic networks, we used the latest update of the Plant Metabolic Network Database (PMND)^24^, which includes 70 plant species classified into the following four major groups in the green plant lineage (with the number of species in each lineage shown in parentheses): green algae (6), early land plants (2) and angiosperms, which are further subdivided into monocots (17) and eudicots (45)^25^. The analysis was performed using an enzyme/reaction-centric network assembled from the full metabolic pathways. The number of plants included in each main evolutionary group is shown in Table S1.

In addition, we assembled networks downloaded from the SignaLink 2.0 database corresponding to signalling pathways from three organisms, namely, *C. elegans, D. melanogaster* and *H. sapiens* ^26^. The statistics for the constructed signal networks are shown in Table S2. The signal proteins in these pathways are classified into seven major pathways that contain all main developmental signalling mechanisms, namely, the Notch, NHR (nuclear hormone receptor), RTK (including EGF/MAPK and IGF/insulin), TGF-B, Wingless/Wnt, Hedgehog and JAK/STAT pathways.

### Fraction and enrichment of control categories for each functional metabolic reaction class

**Figure 4:**
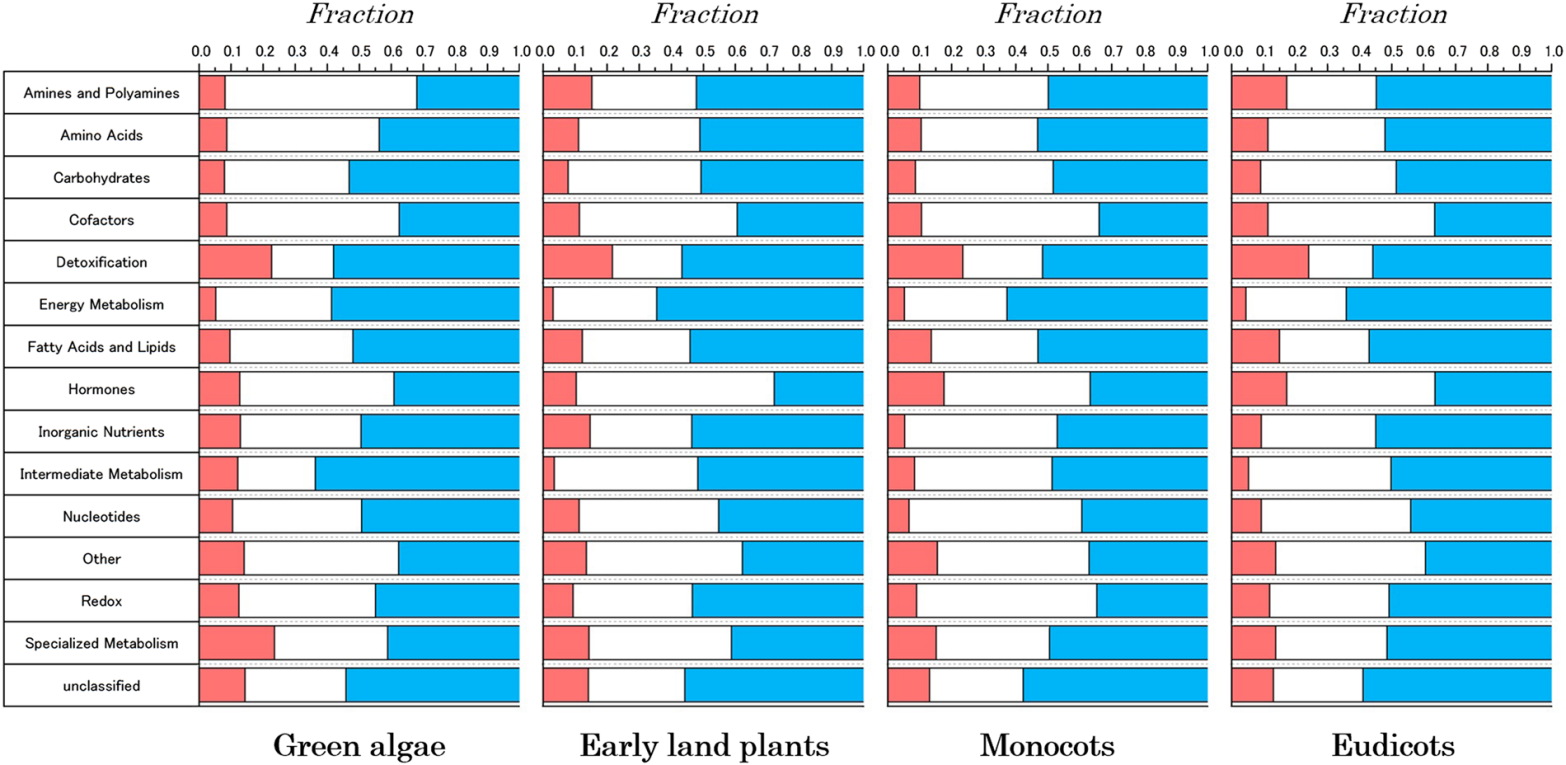
Fraction of enzymes in each control category computed for all plant species in each major evolutionary group and classified into specific functional classes of enzymes. The red fraction indicates the critical nodes, the white fraction denotes the intermittent nodes, and the blue fraction denotes the redundant nodes.

The algorithm was able to identify the controllability categories for each analysed metabolic network. The fraction of metabolic reactions in each control category computed for all plant species in each major evolutionary group and classified into specific functional classes of enzymes showed a heterogeneous distribution. Specifically, some pathways contained a larger fraction of critical nodes (e.g., detoxification), whereas other pathways were largely depleted of critical controllers (e.g., energy metabolism and intermediate metabolism; see Fig. 4). This tendency was largely conserved across evolutionary groups, from green algae to eudicots. However, there are some exceptions, such as specialised metabolism; for this pathway, a large fraction of critical nodes was identified in green algae, but this fraction progressively decreased over evolutionary time. In general, for all examined functional classes, the smallest and largest fractions of nodes corresponded to the critical and redundant nodes, respectively.

**Figure 5:**
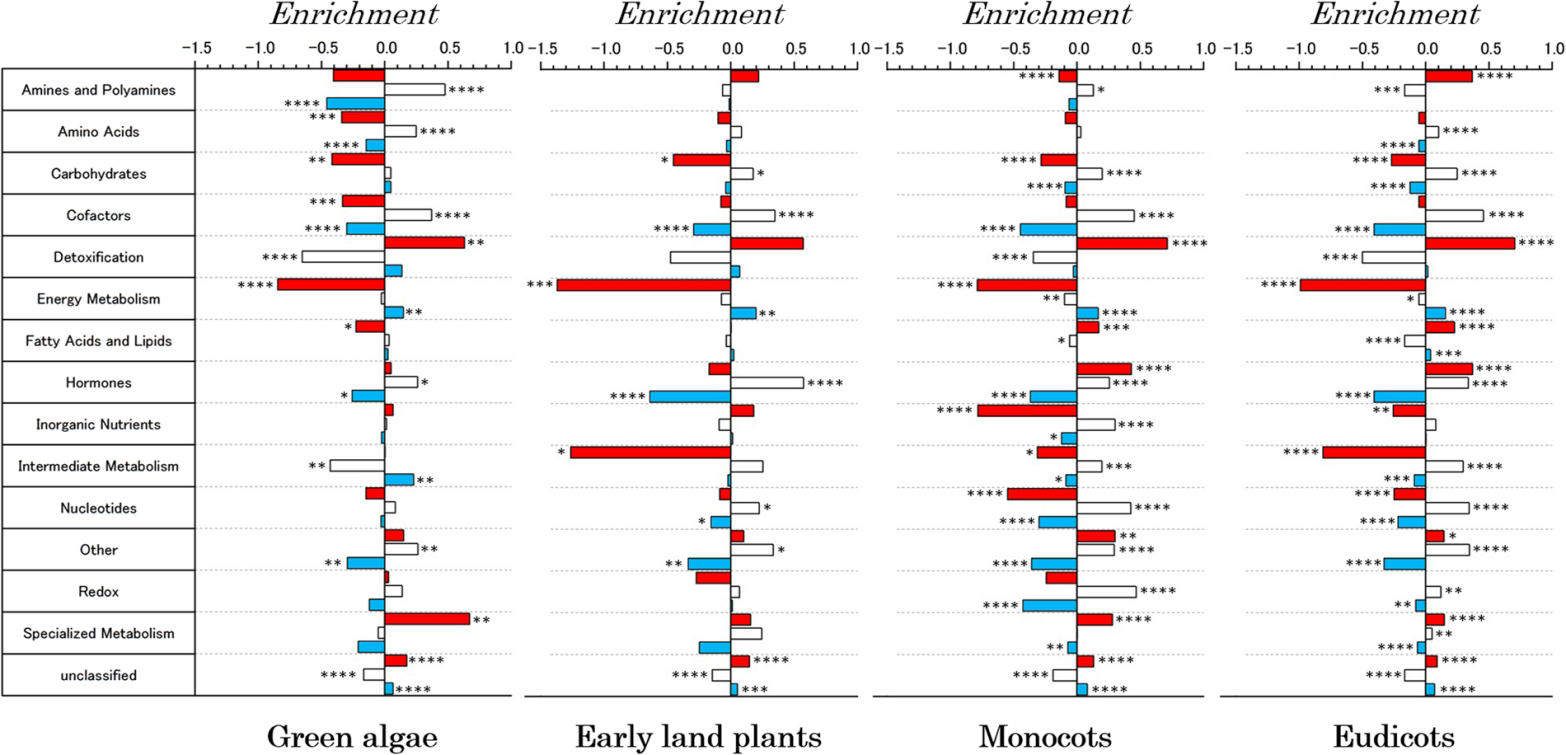
Enrichment or depletion of metabolic enzymes associated with each control category and further classified into functional pathways in each lineage-specific group. The statistical significance was analysed using Fischer’s exact test, and the corresponding two-tailed p-value is shown next to each pathway with the following notations: *p ≤ 0.05, **p ≤ 0.01, ***p ≤ 0.001, and ****p ≤ 0.0001. The colours provide the same information as in Fig. 4.

We then computed the enrichment or depletion of metabolic reactions associated with each control category and further classified these into functional pathways in each lineage-specific group. The results, which are shown in Fig. 5, indicated that the enrichment of controllability categories for functional classes of enzymes was largely conserved across major plant lineages from algae to angiosperms. However, some tendencies regarding the enrichment and depletion of control categories in each functional class were observed. For example, energy metabolism was largely depleted of critical controllers, whereas the detoxification pathway was largely enriched with critical controllers. Moreover, specialised metabolism also showed positive enrichment encompassing all major plant lineages. The highest enrichment of the redundant control category was also observed in energy metabolism. In contrast, nucleotide metabolism was found to be largely enriched with intermittent controllers. The statistical significance of each enrichment, which was assessed by computing the corresponding two-tailed p-value using Fisher’s exact test (see the Methods section for details), is shown in the figure. Furthermore, the fractions of nodes classified into each control category for each analysed plant species are shown in the Supplementary Information (Figs S1–S2). We observed that the fractions were conserved not only within each major plant lineage but also across evolutionary time. The analysis of the enrichment of control categories across functional classes for each individual plant species is shown in the Supplementary Information (Figs S3–S11).

### Fraction and enrichment of control categories of proteins in signal networks and their functional pathways

To further apply the proposed algorithm to real biological systems, we assembled networks downloaded from the SignaLink 2.0 database corresponding to signalling pathways from three organisms, specifically, *C. elegans, D. melanogaster* and *H. sapiens*, and then analysed these three networks in our study. First, we assembled signalling interactions representing experimentally verified physical interactions between molecules that have been classified as (1) directed protein-protein interactions and (2) transcriptional interactions. The latter represents physical protein-DNA binding interactions predicted based on promoter-gene data. Moreover, (3) a combined dataset consisting of both sets of collected data was also prepared.

**Figure 6:**
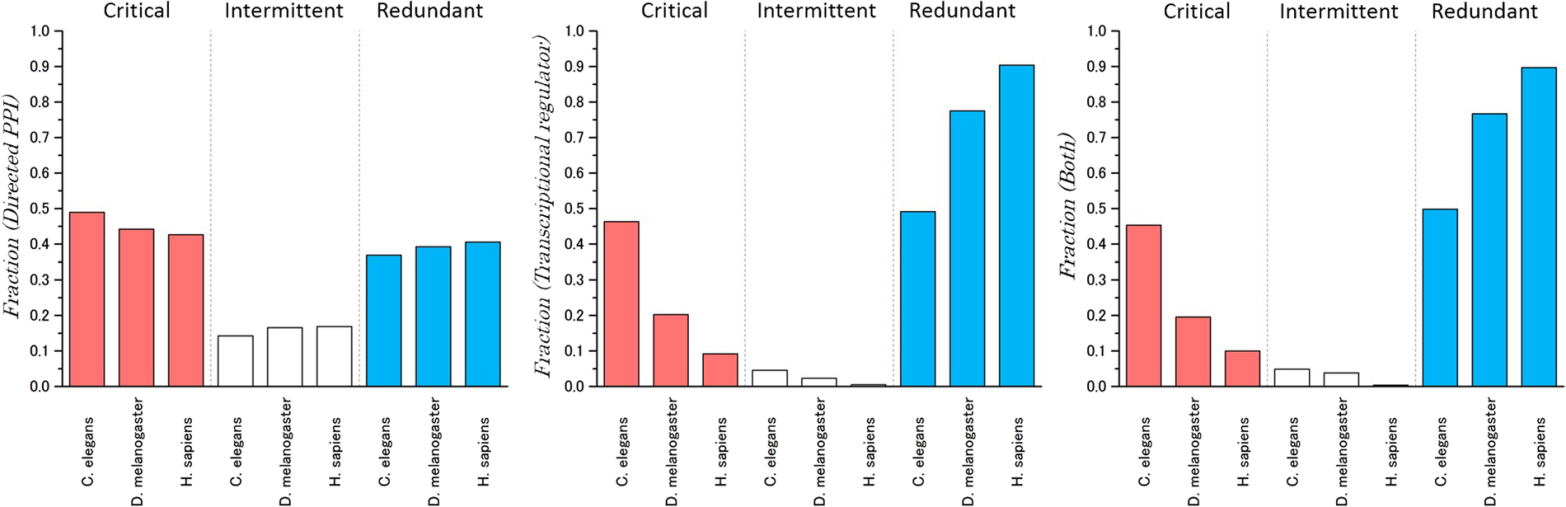
Classification of proteins in all analysed signalling networks of the three organisms into control categories. The colours provide the same information as in Fig. 4.

The fraction of control categories was then computed for each of the assembled networks for the three organisms. The results show that for all three signalling networks, the fraction of critical nodes decreased with increasing organism complexity, i.e., from *C. elegans* to *H. sapiens* (see Fig. 6). Second, the fraction of critical nodes was similar to that of redundant nodes in directed PPI networks. However, a striking difference was observed in the fraction of intermittent nodes. The results of the combined networks showed that the intermittent nodes constituted the smallest fraction in all of the organisms, particularly *H. sapiens* (Fig. 6). This finding highlights the structural differences between metabolic pathways and signalling networks.

**Figure 7:**
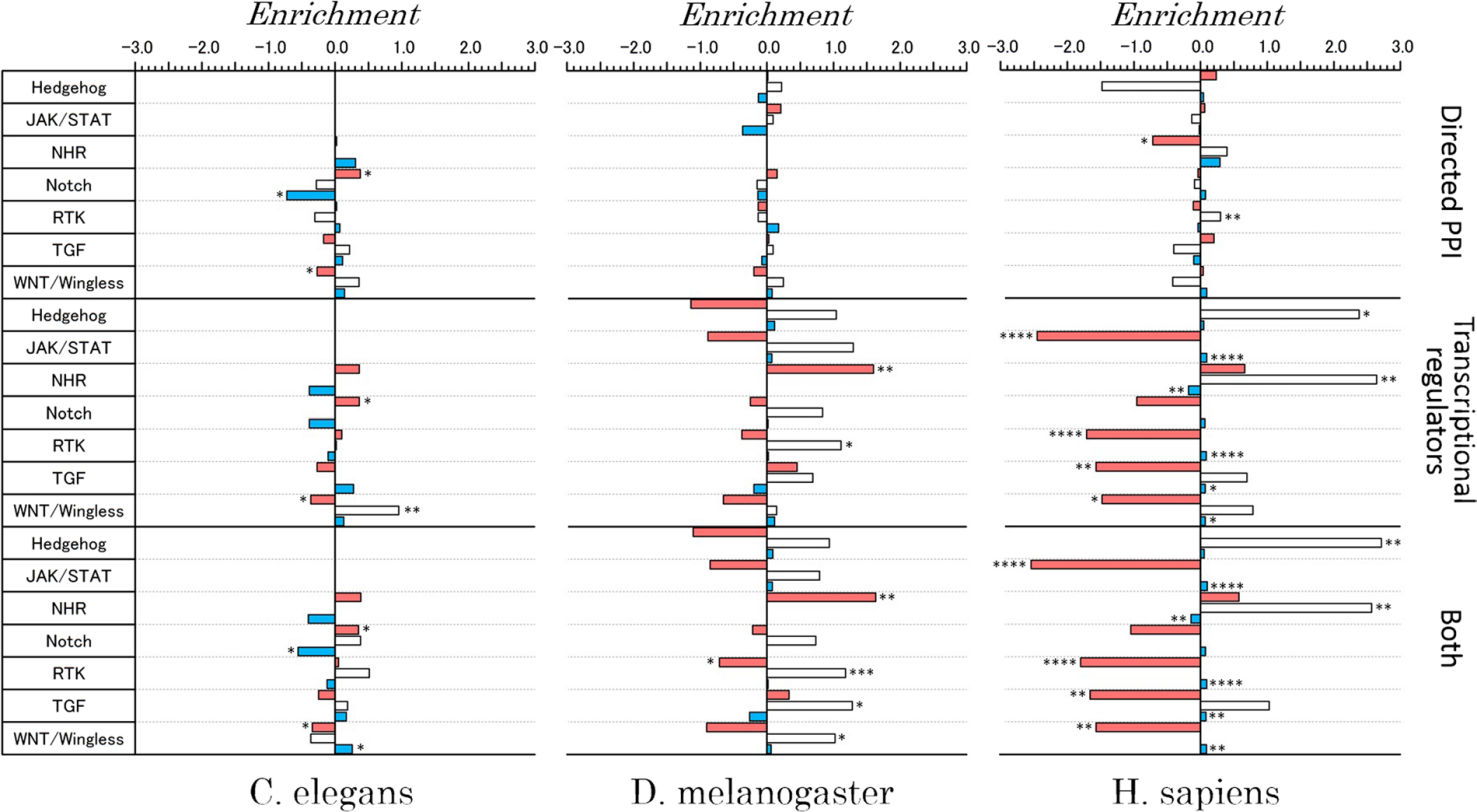
Enrichment or depletion of proteins associated with each control category and further classified into functional signalling pathways in each organism. The statistical significance was analysed using Fischer’s exact test, and the corresponding two-tailed p-value is shown next to each pathway with the following notations: *p ≤ 0.05, **p ≤ 0.01, ***p ≤ 0.001, and ****p ≤ 0.0001. The colours denote the same information as in Fig. 4.

The subsequent enrichment analysis of each major functional pathway showed that most of the pathways were depleted of critical nodes (see Fig. 7), and this finding was obtained due to the existence of few star-type protein hubs, which perform critical roles connecting and controlling multiple molecules (see Fig. 2). An exception was found for the NHR pathway, which showed an enrichment of critical control nodes. The fraction of intermittent nodes was largely enriched in several signalling pathways related to the transcriptional networks of *D. melanogaster* and *H. sapiens*. The Hedgehog and NHR pathways showed the greatest enrichment of intermittent nodes in the combined network. The statistical significance, which was determined based on the two-tailed p-value computed using Fischer’s exact test, is shown in the figure.

## Discussion

In this work, we propose an efficient algorithm for analysing controllability in directed biological networks using the MDS approach. The existing algorithms require solving the MDS problem *n* times, where *n* is the number of nodes^16^, which makes the application of existing algorithms to large and dense biological networks unpractical. In contrast, the proposed algorithm performs efficient graph reduction in a pre-processing step, which aids the identification of a large fraction of critical and redundant controllers and results in a notable simplification of the network. The resulting network is therefore smaller, making it easier to detect the remaining unclassified nodes.

The computational results obtained using artificially constructed scale-free directed networks showed that the proposed algorithm solves the control category problem at a speed up to 176-fold faster than that achieved with existing algorithms. More importantly, the algorithm is able to solve networks consisting of up to 65,000 nodes, indicating that it is suitable for the analysis of genome-scale biological networks.

The algorithm was applied to directed biological networks, such as metabolic pathways and signalling pathways. The analysis of the results revealed that although most control categories are largely conserved across evolutionary time, from green algae and early plants to modern angiosperm plant lineages, some specific metabolic functions tend to have more critical nodes than others. Therefore, we were able to associate biological functions to certain control categories. For example, detoxification reaction classes show a high enrichment of critical nodes. In addition, the detoxification pathway contains a class of proteins and enzymes specialised in degradation and/or elimination of endogenous and exogenous toxins^27^, and the concentration of these enzymes represents a response to an increase of toxin stimulation. Our analysis showed that these enzymes perform structural control functions in the network. In contrast, the genes associated with specialised metabolism encode enzymes that reflect evolutionary mutations that occurred in response to specific environmental changes^28,29^. Our results show that a significant fraction of the enzymes in the specific functional category also participates in critical network control.

Signalling pathways show a unique network control category distribution. In fact, as shown in Fig. 6, signalling pathways include a small fraction of intermittent nodes, and this is a consequence of the structural differences between metabolic and signal networks. Among the functional pathways, the NHR pathway showed a conserved enrichment of critical control proteins (Fig. 7). Signal proteins associated with the NHR pathway represent a class of ligand-activated proteins that perform on-off transcriptional switches when bound to specific DNA sequences within the cell nucleus. These functional switches play roles in the control of the development, growth and differentiation of skin, bone and behavioural areas in the brain, the determination of sex, the continual regulation of reproductive tissues, and the regulation of cell metabolism.

We believe that analysing the controllability of biological networks is a useful technique for identifying critical control-related molecules associated with not only specific biological functions but also human disorders. In future, we encourage further studies on the identification of critical disease-related proteins in directed biological networks.

## Methods

### Datasets

The data used in this study correspond to metabolic pathways of 70 plant species downloaded from the Plant Metabolic Network Database version 16.0. These species were classified into major plant lineages as follows: six species correspond to green algae, two species are early land plants, 17 species are monocots, and 45 species are eudicots^25^. The analysis was performed using an enzyme/reaction-centric network assembled from the full metabolic pathways. The bipartite network is therefore transformed into a unipartite network in which nodes corresponds to the enzymes. The statistics for the assembled networks are shown in Table S1 in the Supplementary Information (SI). The analysed signalling pathways of directed protein-protein and transcriptional interactions corresponded to those from three organisms, namely, C*. elegans, D. melanogaster* and *H. sapiens* ^26^. The data were collected from the SignaLink 2.0 database. The statistics for the signalling pathway networks are shown in Table S2 in the SI.

### Algorithm for the efficient computation of critical and redundant dominating subsets in directed complex networks

The combination of the propositions introduced in the Results section led to the development of the following algorithm for the efficient computation of control categories in large directed complex networks (see Fig. 1 for an illustration of the algorithm steps):

1. The critical proposition 2.3 is applied to each node.
2. The critical proposition 2.4 is applied to each unclassified node, and the incoming links to the newly identified critical nodes are deleted.
3. The redundant proposition 1 is applied to each unclassified node, and the incoming links to the newly identified redundant nodes are deleted.
4. If Steps 2 and 3 involved the deletion of one or more links, propositions 2.4 and 1 in Steps 2 and 3, respectively, are re-applied.
5. The simplified MDS problem for the resulting network is applied after adding information obtained from Steps 1~4 into the ILP.
6. After an MDS solution is obtained, the control category algorithm for critical and redundant roles is applied to classify the remaining nodes as follows:

6.1 The critical algorithmic procedure is applied to each node that belongs to the MDS^16^ and has not already been identified as critical to determine its critical role.
6.2 The redundant algorithmic procedure is applied to each node that does not belong to the MDS^16^ to determine its redundant role.

For artificial network analysis, the formalised Integer Linear Programming (ILP) problem was solved using the GNU Linear Programming Solver (glpsol). For data analysis, the ILP problem was solved using the IBM ILOG CPLEX Optimiser package version 12.0.

### Enrichment analysis

To assess the extent to which each control category is present in each metabolic and signalling pathway, we used the following enrichment factor. We first computed the fraction of enzymes or proteins identified for a given control category (S) and existing in a given enzyme functional class or pathway (P) ${N}_{S}^{P}$ divided by the total number of enzymes or proteins identified for the given category (S) *N*_*S*_, yielding ${f}_{S}^{P}={N}_{S}^{P}/{N}_{S}$. We then calculated the fraction of enzymes or proteins that are located in a given enzyme functional class or pathway *N* ^*P*^ divided by the total number of nodes (enzymes or proteins) *N* in a given metabolic network or signal network, i.e., ${f}^{P}={N}^{P}/N$. The enrichment factor for a control category (S) in a given pathway (P) was then computed as ${E}_{S}^{P}=\,\mathrm{ln}({f}_{S}^{P}/{f}^{P})$. We then defined a contingency table and evaluated the statistical significance of the enrichment or depletion of the identified critical, intermitted and redundant enzymes or proteins in each functional class or pathway using Fisher’s exact test. The two-tailed exact p-values are denoted in each figure using the following notations: *p ≤ 0.05, **p ≤ 0.01, ***p ≤ 0.001, and ****p ≤ 0.0001.

In the analysis of signalling networks, only one organism was included in each enrichment computation. The computation of the enrichment of a control category (S) in a given enzyme functional class or pathway (P) for all organisms included in a major plant lineage involved ${N}_{S}^{P}$, *N*_*S*_, *N* ^*P*^, and *N*, where each variable has the same meaning as defined above but aggregates the total number of counts observed in each major lineage.

### Data Availability

The metabolic pathways were downloaded from the publicly available Plant Metabolic Network Database Version 12.0 (PMN) www.plantcyc.org. The signal pathways were downloaded from the publicly available SignalLink 2.0 Database http://signalink.org/. We did not perform biological experiments, therefore, we did not generate any new biological dataset in this study. All the computational results are presented in figures and included in this manuscript and in the Supplementary Information that accompanies this paper.

## Electronic supplementary material

Supplementary Information
