# Supplementary material for: Critical controllability analysis of directed biological networks using efficient graph reduction
Source: Sci Rep. 2017 Oct 30;7:14361. doi: 10.1038/s41598-017-14334-8 (PMC5662738; doi:10.1038/s41598-017-14334-8)
Supplement: Supplementary file 1 — Supplementary Information [file 41598_2017_14334_MOESM1_ESM.doc]

**Supplementary Information**

**Critical controllability analysis of directed biological networks
using efficient graph reduction**

Masayuki Ishitsuka, Tatsuya Akutsu and Jose C. Nacher

**Contents**

Supplementary Figures SI1-SI11

Supplementary Tables SI1 and SI2.


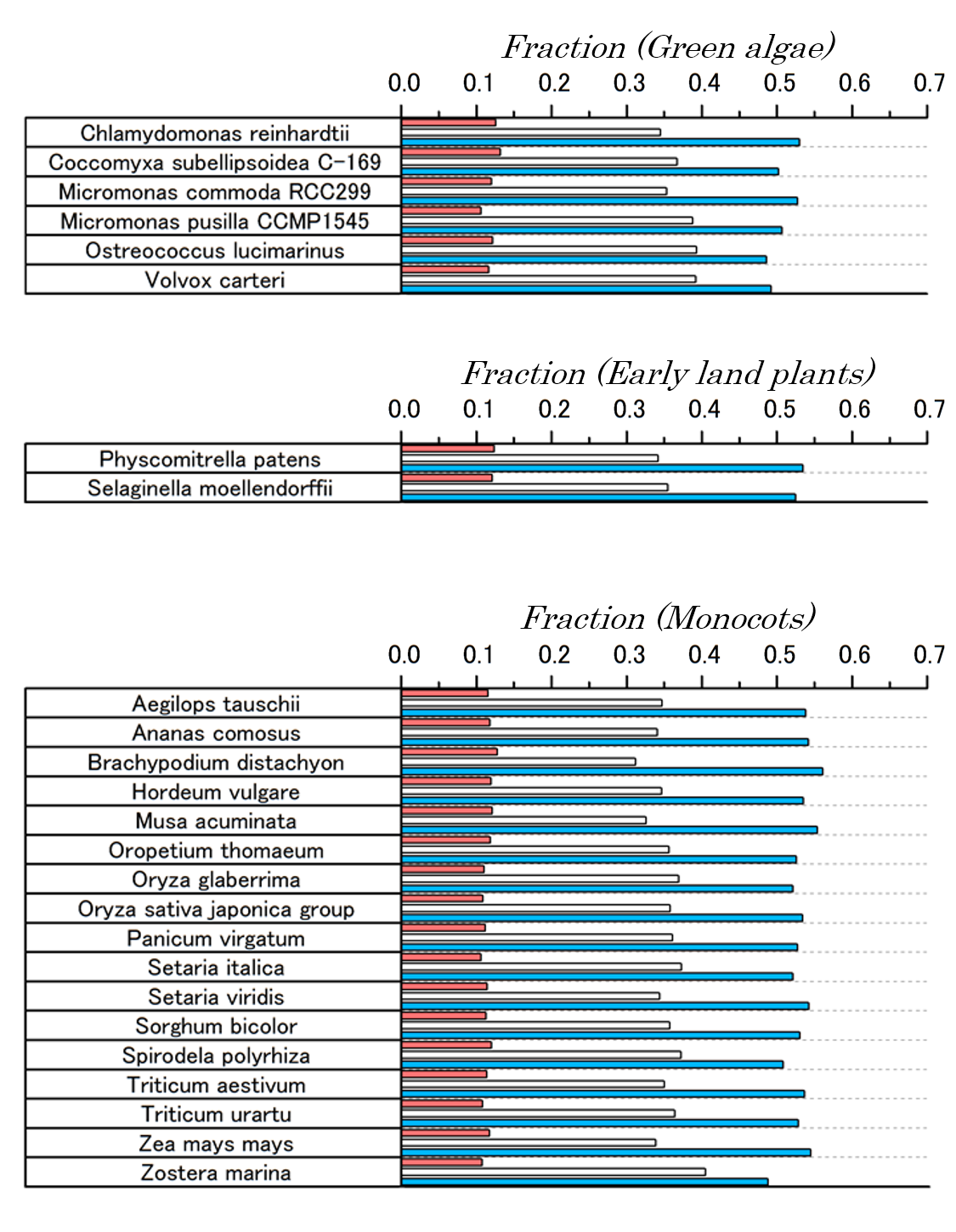


Fig. SI1: Fraction of nodes from each individual plant organism (from the green algae, early land plant and monocot major evolutionary groups) classified into critical, intermittent and redundant control categories


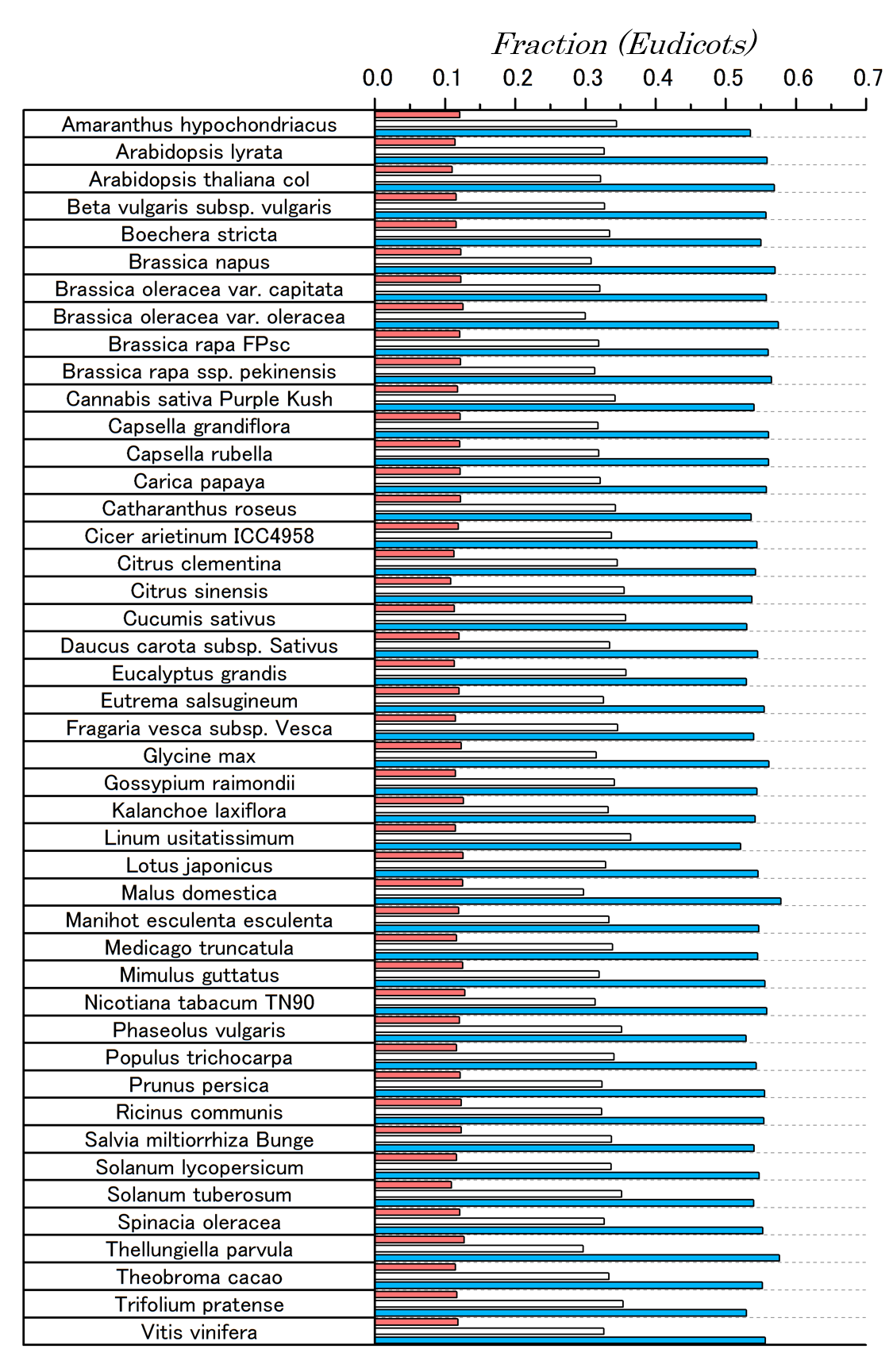
Fig. SI2: Fraction of nodes from each individual plant organism (from the eudicot evolutionary group) classified into critical, intermittent and redundant control categories.


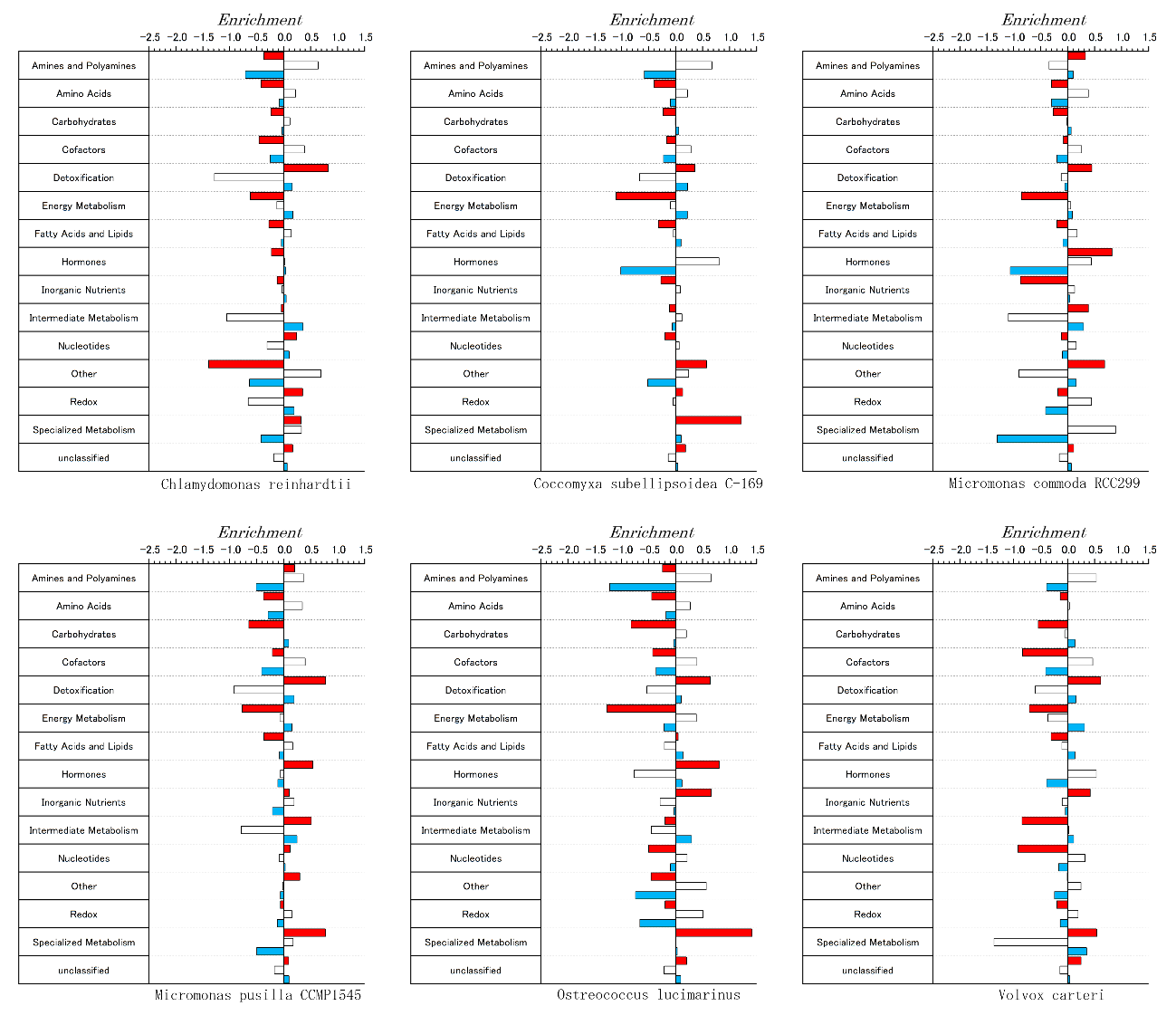


Fig. SI3 Enrichment results for each individual plant organism in the green algae evolutionary group.


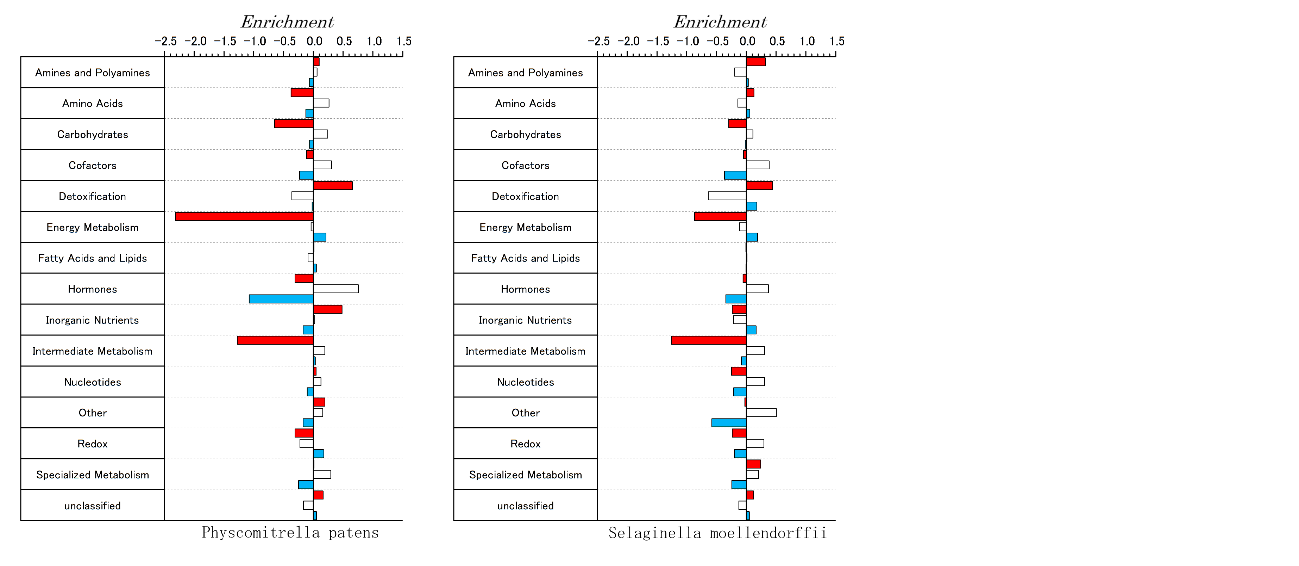


Fig. SI4 Enrichment results for each individual plant organism in the early land plant evolutionary group.


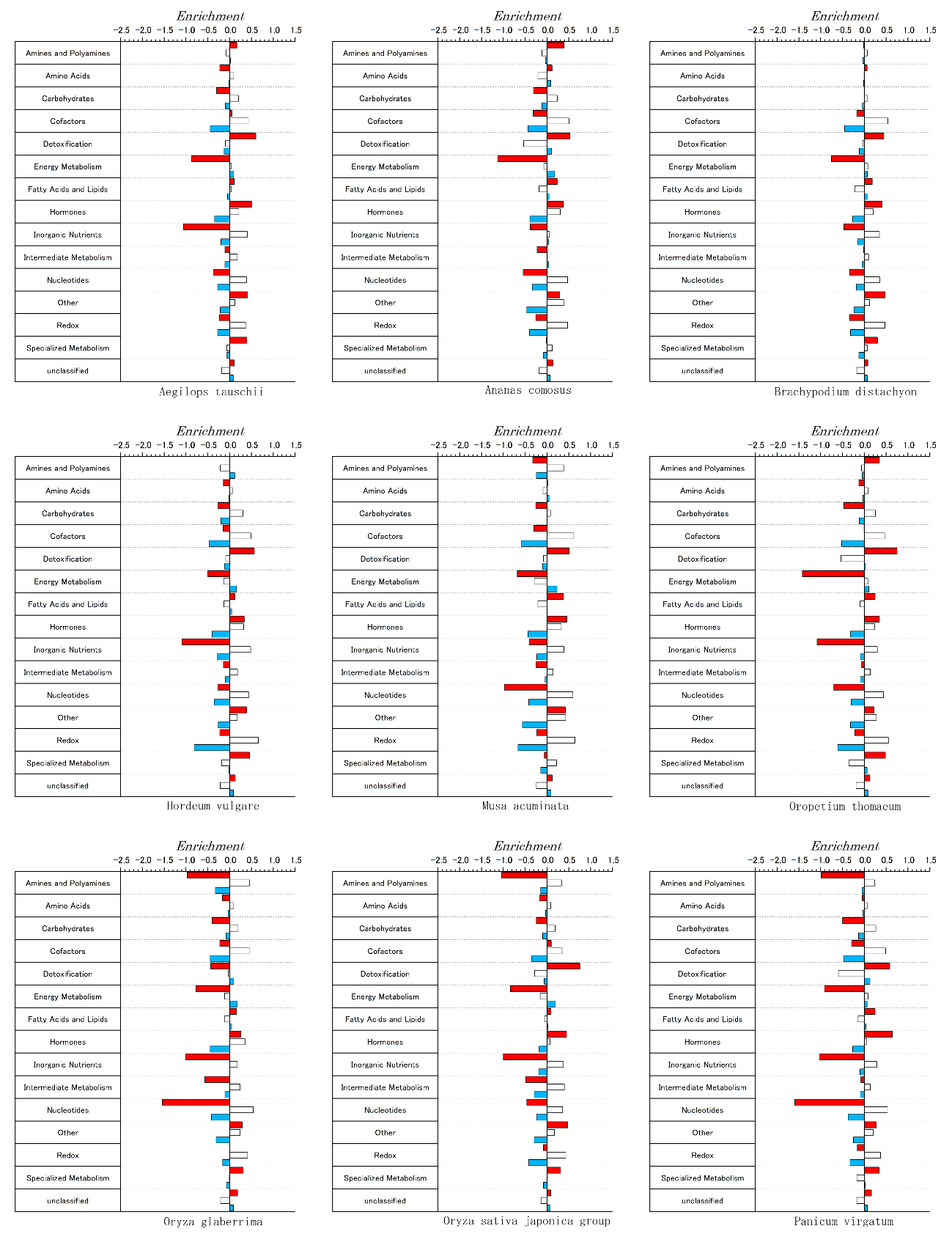


Fig. SI5 Enrichment results for each individual plant organism in the monocot group (part 1).


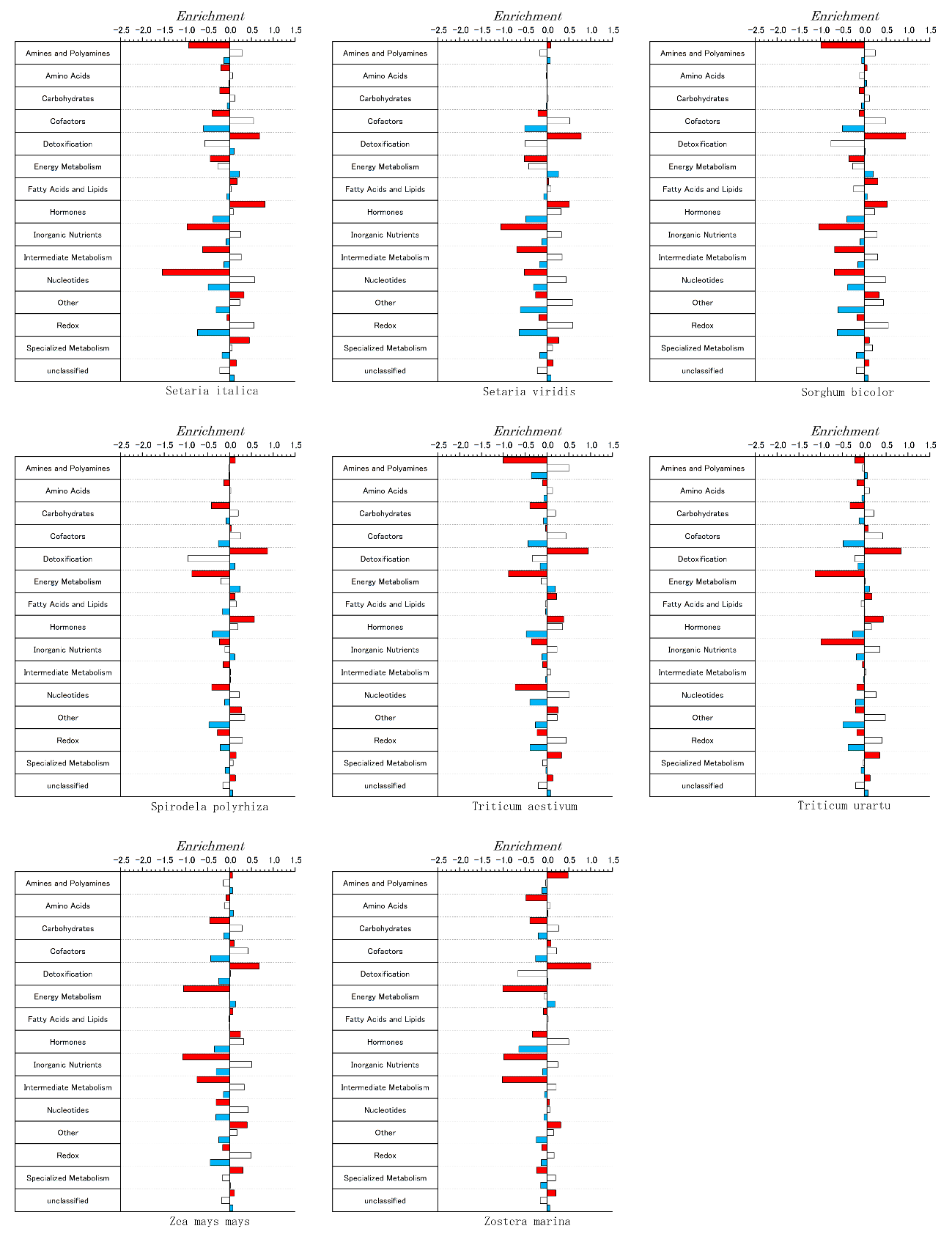


Fig. SI6 Enrichment results for each individual plant organism in the monocot group (part 2).


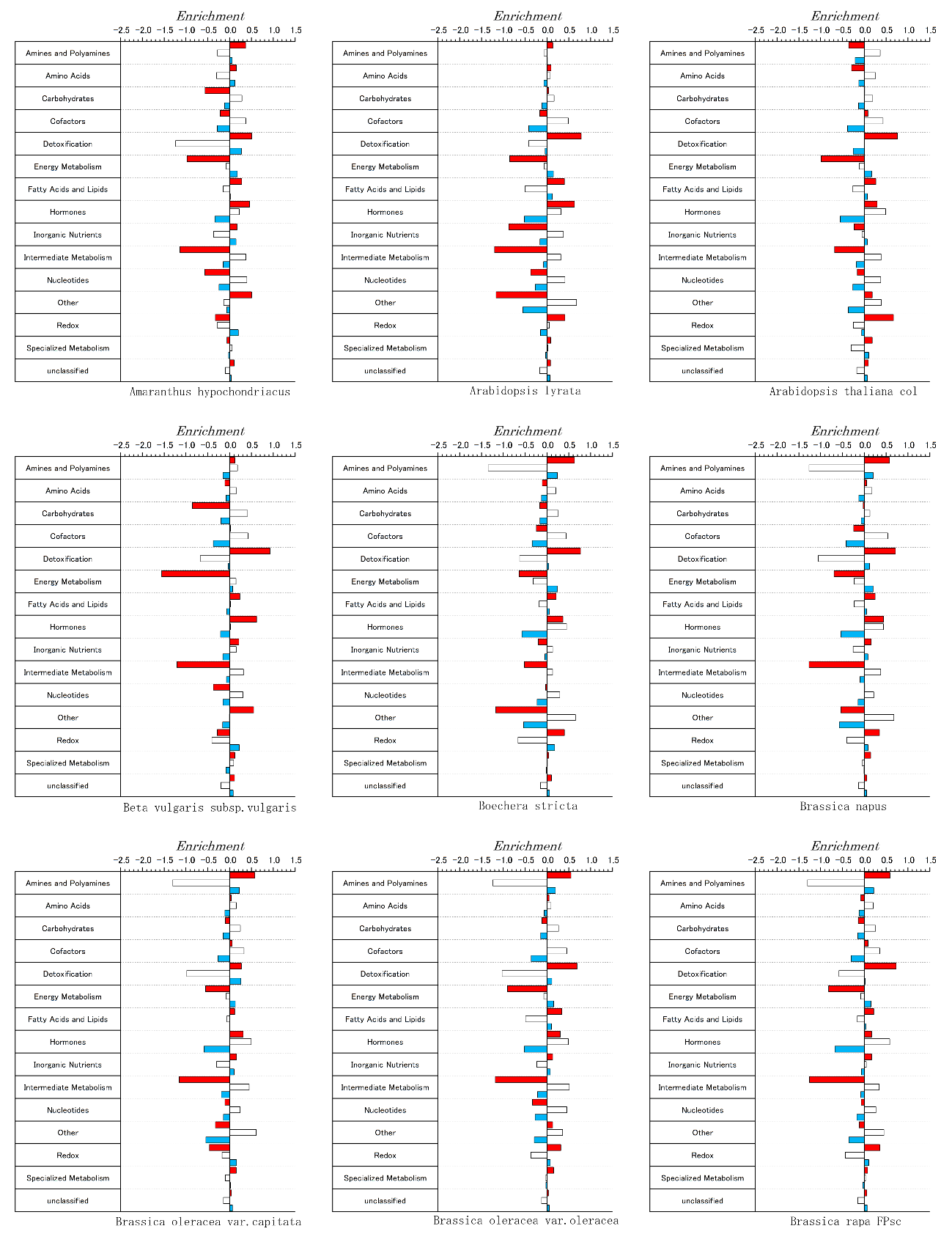


Fig. SI7 Enrichment results for each individual plant organism in the eudicot group (part 1).


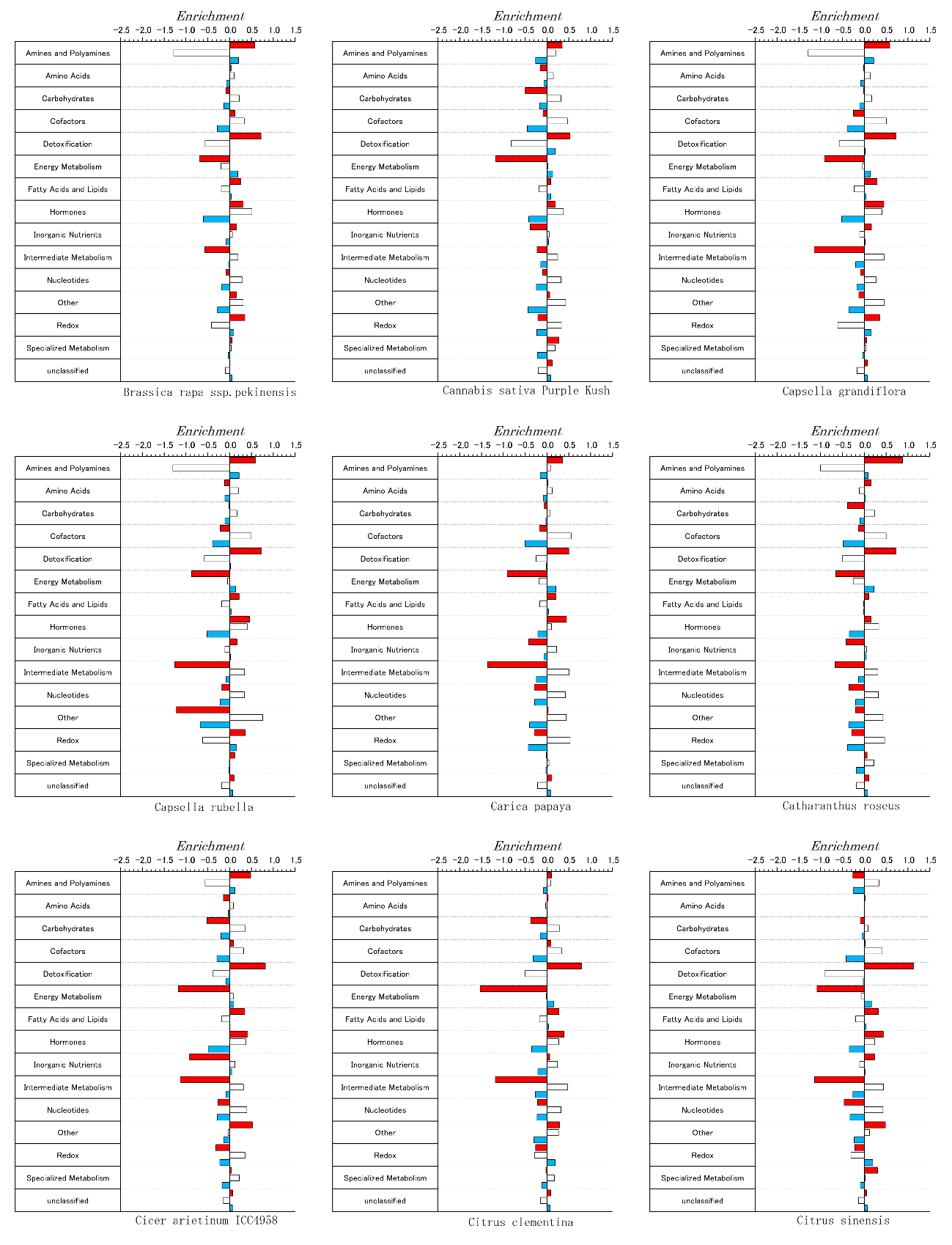


Fig. SI8: Enrichment results for each individual plant organism in the eudicot group (part 2).


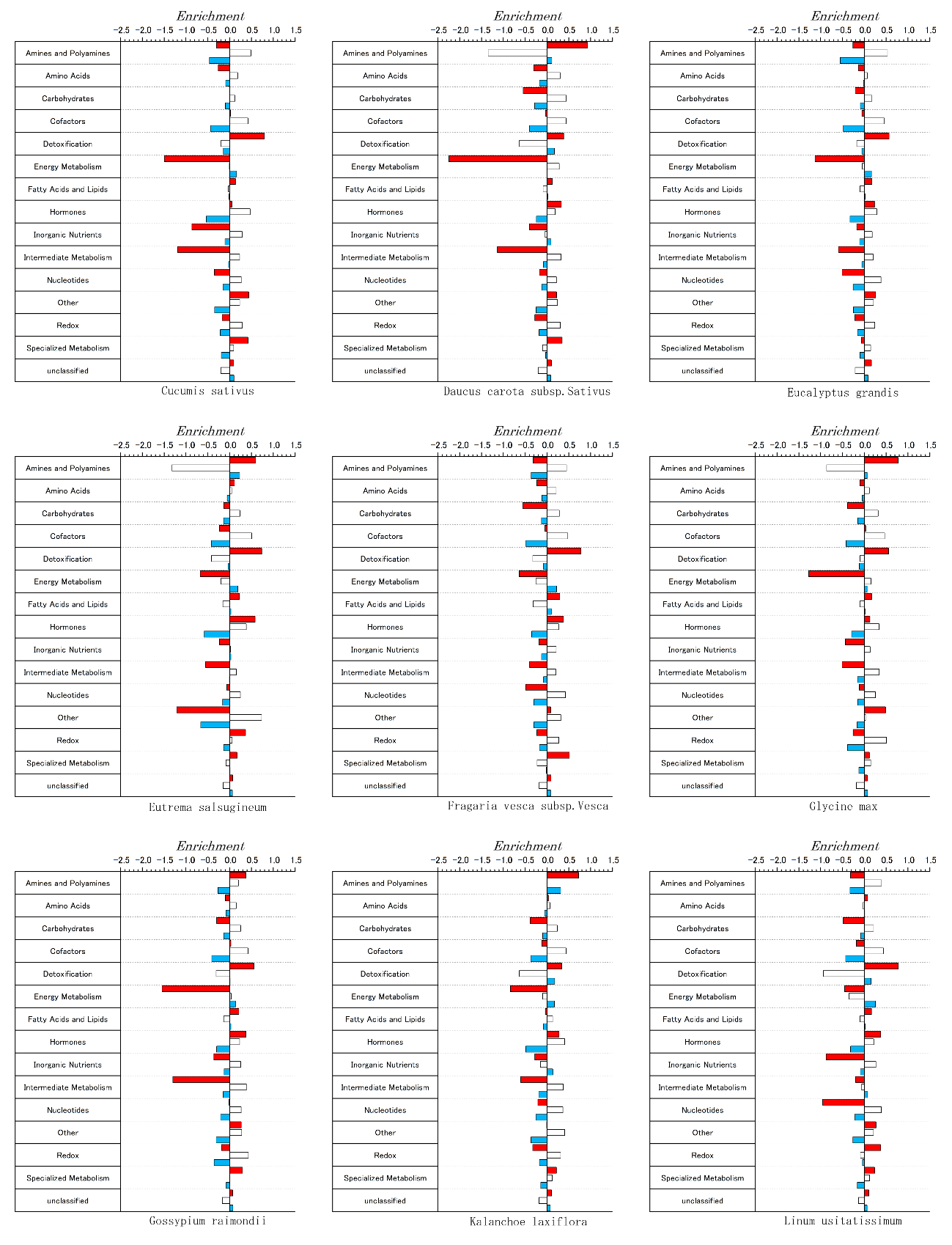


Fig. SI9: Enrichment results for each individual plant organism in the eudicot group (part 3).


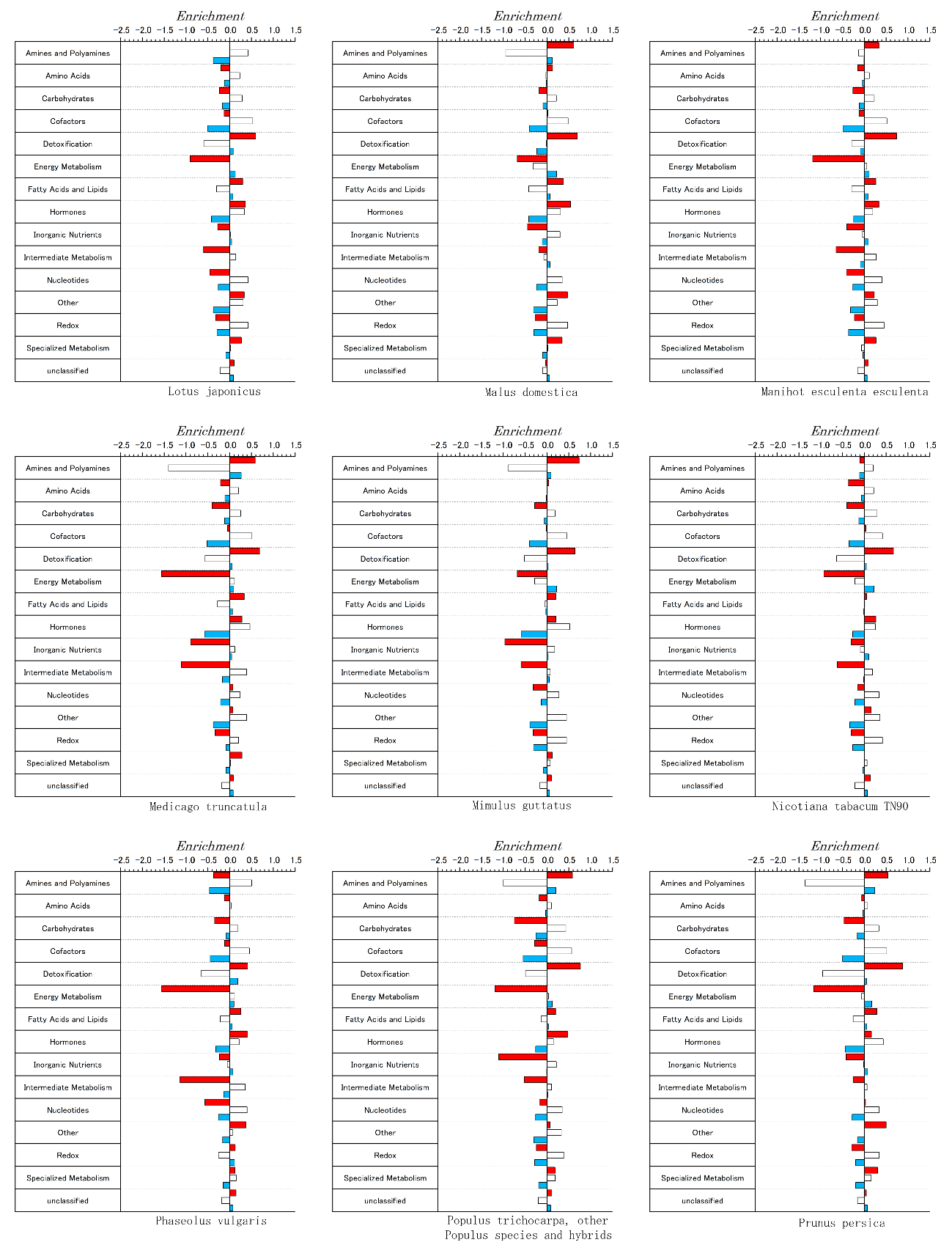


Fig. SI10: Enrichment results for each individual plant organism in the eudicot group (part 4).


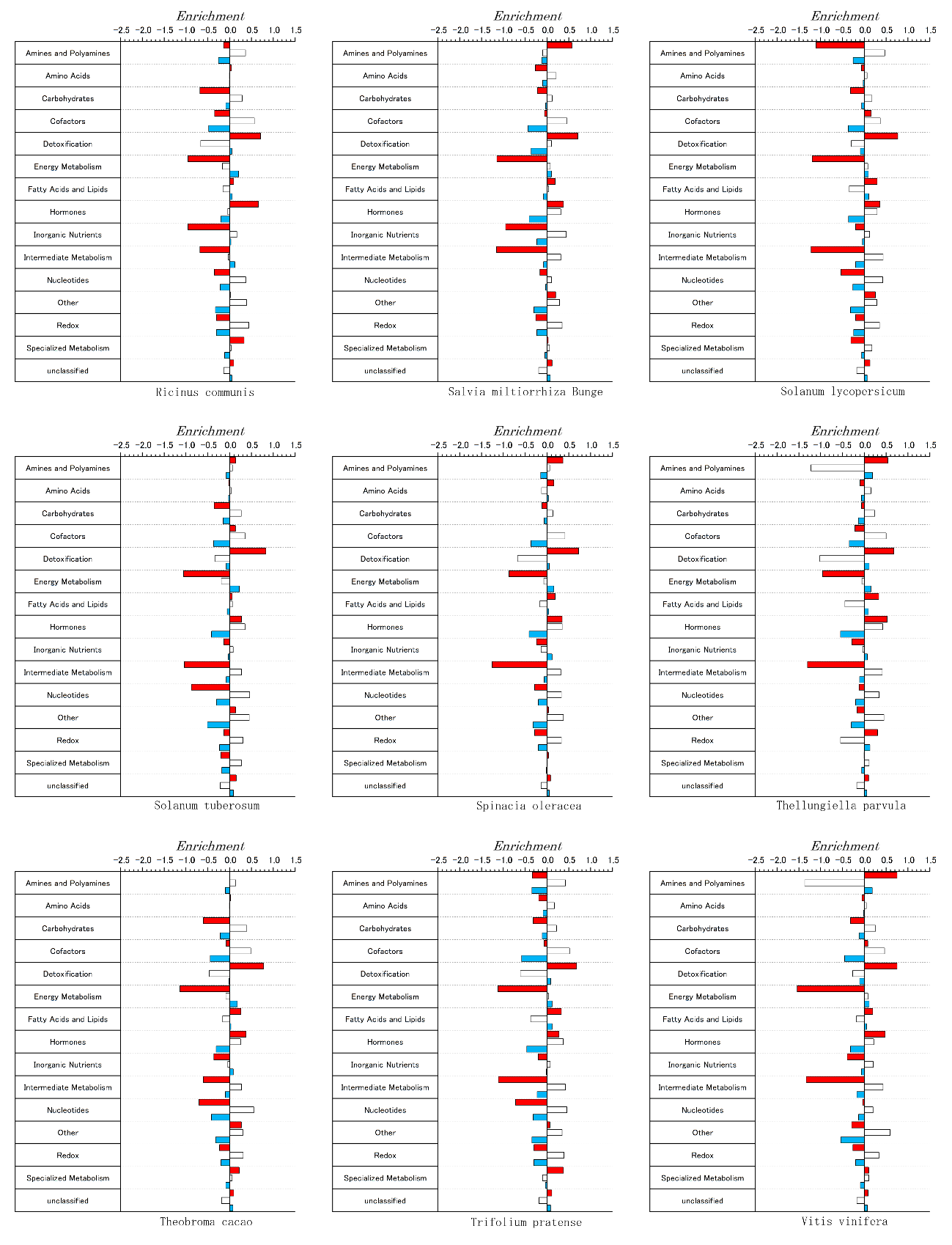


Fig. SI11: Enrichment results for each individual plant organism in the eudicot group (part 5).

| **Organism** | **Group** | **#Node** | **#Link** |
| --- | --- | --- | --- |
| **Aegilops tauschii** | Monocots | 1837 | 24134 |
| **Amaranthus hypochondriacus** | Eudicots | 1764 | 25113 |
| **Ananas comosus** | Monocots | 1777 | 24602 |
| **Arabidopsis lyrata** | Eudicots | 1925 | 27294 |
| **Arabidopsis thaliana col** | Eudicots | 2067 | 29710 |
| **Beta vulgaris subsp. vulgaris** | Eudicots | 1861 | 26193 |
| **Boechera stricta** | Eudicots | 1882 | 25814 |
| **Brachypodium distachyon** | Monocots | 1828 | 24373 |
| **Brassica napus** | Eudicots | 1997 | 28554 |
| **Brassica oleracea var. capitata** | Eudicots | 1858 | 24302 |
| **Brassica oleracea var. oleracea** | Eudicots | 1975 | 27148 |
| **Brassica rapa FPsc** | Eudicots | 1906 | 26170 |
| **Brassica rapa ssp. pekinensis** | Eudicots | 1897 | 26005 |
| **Cannabis sativa Purple Kush** | Eudicots | 1888 | 26276 |
| **Capsella grandiflora** | Eudicots | 1869 | 25391 |
| **Capsella rubella** | Eudicots | 1882 | 25884 |
| **Carica papaya** | Eudicots | 1901 | 25661 |
| **Catharanthus roseus** | Eudicots | 2021 | 28258 |
| **Chlamydomonas reinhardtii** | Green algae | 1570 | 26993 |
| **Cicer arietinum ICC4958** | Eudicots | 2028 | 31713 |
| **Citrus clementina** | Eudicots | 1854 | 25930 |
| **Citrus sinensis** | Eudicots | 1879 | 25761 |
| **Coccomyxa subellipsoidea C-169** | Green algae | 1437 | 21577 |
| **Cucumis sativus** | Eudicots | 1795 | 24816 |
| **Daucus carota subsp. Sativus** | Eudicots | 1841 | 24571 |
| **Eucalyptus grandis** | Eudicots | 1863 | 25606 |
| **Eutrema salsugineum** | Eudicots | 1879 | 25627 |
| **Fragaria vesca subsp. Vesca** | Eudicots | 1850 | 26036 |
| **Glycine max** | Eudicots | 2016 | 30123 |
| **Gossypium raimondii** | Eudicots | 1898 | 26760 |
| **Hordeum vulgare** | Monocots | 1786 | 23411 |
| **Kalanchoe laxiflora** | Eudicots | 1872 | 25296 |
| **Linum usitatissimum** | Eudicots | 1956 | 28062 |
| **Lotus japonicus** | Eudicots | 1916 | 25766 |
| **Malus domestica** | Eudicots | 1994 | 28963 |
| **Manihot esculenta esculenta** | Eudicots | 1916 | 26185 |
| **Medicago truncatula** | Eudicots | 1988 | 28059 |
| **Micromonas commoda RCC299** | Green algae | 1341 | 18589 |
| **Micromonas pusilla CCMP1545** | Green algae | 1293 | 17478 |
| **Mimulus guttatus** | Eudicots | 1889 | 25694 |
| **Musa acuminata** | Monocots | 1813 | 25506 |
| **Nicotiana tabacum TN90** | Eudicots | 1920 | 27515 |
| **Oropetium thomaeum** | Monocots | 1719 | 22716 |
| **Oryza glaberrima** | Monocots | 1808 | 23825 |
| **Oryza sativa japonica group** | Monocots | 1907 | 27034 |
| **Ostreococcus lucimarinus** | Green algae | 1217 | 16133 |
| **Panicum virgatum** | Monocots | 1970 | 28690 |
| **Phaseolus vulgaris** | Eudicots | 1882 | 25117 |
| **Physcomitrella patens** | Early land plants | 1764 | 29459 |
| **Populus trichocarpa, other Populus species and hybrids** | Eudicots | 1943 | 27897 |
| **Prunus persica** | Eudicots | 1895 | 26156 |
| **Ricinus communis** | Eudicots | 2274 | 37189 |
| **Salvia miltiorrhiza Bunge** | Eudicots | 1846 | 24043 |
| **Selaginella moellendorffii** | Early land plants | 1697 | 24283 |
| **Setaria italica** | Monocots | 1838 | 24837 |
| **Setaria viridis** | Monocots | 1845 | 25489 |
| **Solanum lycopersicum** | Eudicots | 1950 | 26834 |
| **Solanum tuberosum** | Eudicots | 1910 | 25484 |
| **Sorghum bicolor** | Monocots | 1801 | 25292 |
| **Spinacia oleracea** | Eudicots | 1796 | 26009 |
| **Spirodela polyrhiza** | Monocots | 1685 | 22330 |
| **Thellungiella parvula** | Eudicots | 1891 | 26275 |
| **Theobroma cacao** | Eudicots | 1902 | 26781 |
| **Trifolium pratense** | Eudicots | 1951 | 26841 |
| **Triticum aestivum** | Monocots | 2030 | 29182 |
| **Triticum urartu** | Monocots | 1880 | 25502 |
| **Vitis vinifera** | Eudicots | 1915 | 26781 |
| **Volvox carteri** | Green algae | 1409 | 20750 |
| **Zea mays mays** | Monocots | 1895 | 26599 |
| **Zostera marina** | Monocots | 1604 | 22297 |

Table SI1 : Plant metabolic networks constructed using data from the Plant Metabolic Network Database PMN 12.0. The table indicates the plant name as well as the number of nodes and links in the main component.

| **Organism** | **Interaction** | **#Node** | **#Link** |
| --- | --- | --- | --- |
| **Caenorhabditis elegans** | Directed protein-protein interactors | 190 | 256 |
|  | Transcriptional regulators | 285 | 400 |
|  | Both | 287 | 430 |
| **Drosophila melanogaster** | Directed protein-protein interactors | 181 | 333 |
|  | Transcriptional regulators | 658 | 5122 |
|  | Both | 660 | 5269 |
| **Homo sapiens** | Directed protein-protein interactors | 570 | 1106 |
|  | Transcriptional regulators | 2318 | 18030 |
|  | Both | 2350 | 21841 |

Table SI2 :. Signal networks constructed using data from the SignaLink 2.0 database. The table shows the organism name, the type of interactions considered and the number of nodes and links in the network.
